# Slag Blended Cement Paste Carbonation under Different CO_2_ Concentrations: Controls on Mineralogy and Morphology of Products

**DOI:** 10.3390/ma13153404

**Published:** 2020-08-01

**Authors:** Wei Liu, Shifa Lin, Yongqiang Li, Wujian Long, Zhijun Dong, Luping Tang

**Affiliations:** 1 Guangdong Provincial Key Laboratory of Durability for Marine Civil Engineering, College of Civil and Transportation Engineering, Shenzhen University, Shenzhen 518060, China; liuwei@szu.edu.cn (W.L.); linshifa2018@email.szu.edu.cn (S.L.); 18503009753@163.com (Y.L.); 2 Institute of Technology for Marine Civil Engineering, Shenzhen Institute of Information Technology, Shenzhen 518172, China; dongzj@sziit.edu.cn; 3 Department of Architecture and Civil Engineering, Division of Building Technology, Chalmers University of Technology, 41296 Gothenburg, Sweden; tang.luping@chalmers.se

**Keywords:** slag, carbonation, CO_2_ concentration, carbonation products, morphology

## Abstract

To investigate the effect of different CO_2_ concentrations on the carbonation results of slag blended cement pastes, carbonation experiments under natural (0.03% CO_2_) and accelerated conditions (3, 20, and 100% CO_2_) were investigated with various microscopic testing methods, including X-ray diffraction (XRD), ^29^Si magic angle spinning nuclear magnetic resonance (^29^Si MAS NMR) and scanning electron microscopy (SEM). The XRD results indicated that the major polymorphs of CaCO_3_ after carbonation were calcite and vaterite. The values of the calcite/(aragonite + vaterite) (c/(a + v)) ratios were almost the same in all carbonation conditions. Additionally, NMR results showed that the decalcification degree of C-S-H gel exposed to 0.03% CO_2_ was less than that exposed to accelerated carbonation; under accelerated conditions, it increased from 83.1 to 84.2% when the CO_2_ concentration improved from 3% to 100%. In SEM observations, the microstructures after accelerated carbonation were denser than those under natural carbonation but showed minor differences between different CO_2_ concentrations. In conclusion, for cement pastes blended with 20% slag, a higher CO_2_ concentration (above 3%) led to products different from those produced under natural carbonation. A further increase in CO_2_ concentration showed limited variation in generated carbonation products.

## 1. Introduction

The durability of reinforced concrete structures has attracted much attention over the past several decades [1,2,3]. One of the most concerning issues is carbonation, which refers to the reaction between CO_2_ and Ca-bearing cement hydration products in concrete that decreases alkalinity and causes precipitation of CaCO_3_ [4,5]. In the presence of moisture and oxygen, the passive film that protects steel rebar is destroyed when the pH falls below 9 [6]. It is well known that carbonation changes the mineral phase and modifies the pore structures and transport properties of ions in concrete [7,8,9]. Calcium silicate hydrate (C-S-H) and portlandite (CH) are the dominating hydration phases in a hydrated cement matrix that reacts with CO_2_ at a volume fraction higher than 70%. Based on previous studies, CH was carbonated to calcite with the release of water, while three kinds of polymorphs (i.e., calcite, aragonite and vaterite) were generated accompanied with silica gel after the carbonation of C-S-H [4,10]. Moreover, the carbonation process also changes the pore structures of the cement matrix due to the different molar volumes of CaCO_3_ over CH and C-S-H [11,12].

Slag, an industrial by-product, has been considered as an excellent admixture used worldwide in concrete constructions [13,14]. In concrete manufactured with slag to supplement Portland cement, the CH generated from cement hydration can be partially consumed, which decreases the alkalinity of the admixture and generates the C-S-H with a Ca/Si ratio of about 1 [15,16]. Besides, with the partial substitution of cement by slag, the usage of cement can be dramatically reduced, which is beneficial for a reduction in CO_2_ emission [17,18]. Additionally, compared with samples made with ordinary Portland cement (OPC), workability can be improved with the supplementation of slag. All of these advantages promote the frequency of using slag as supplementary cementitious material for concrete fabrication.

To evaluate the carbonation resistance of concrete, it is more reliable to conduct carbonation experiments under natural conditions, which is a slow process and requires decades for noticeable ingress from the exposure surface. Therefore, accelerated carbonation is used in laboratory experiments to predict the long-term carbonation performance of concrete under natural conditions, where the carbonation rate is assumed to elevate linearly with the square root of CO_2_ concentration [6]. However, the CO_2_ concentration (vol %) adopted for accelerated carbonation varies from each country: 1–4% CO_2_ in some European countries, 20% in China and 50% in France. Even 100% CO_2_ was used by some researchers to speed up the rate of carbonation [9,19]. Unfortunately, there is still no consensus on CO_2_ concentration selectivity used for accelerated carbonation.

A previous study of carbonation on OPC pastes and fly ash (FA) added pastes under various CO_2_ concentrations revealed that mineral compositions under accelerated carbonation differed from those produced under natural carbonation but were similar to 3% and 20% CO_2_, respectively, and differed further in the experiment using 100% CO_2_ [20]. However, when blended with slag, the mineral compositions of the cement pastes were dramatically changed compared with the OPC and FA blended samples. It is unknown whether this change in mineral composition affects the carbonation results of slag-blended cement-based materials [21,22,23], which should be significant from an engineering point of view due to the high replacement ratio of cement by slag in practical engineering [24,25].

The aim of this study was to investigate changes in mineral composition and morphology of slag blended cement pastes subjected to natural carbonation and accelerated carbonation with different CO_2_ concentrations (3, 20 and 100%). The impact of slag supplementation on variations in CaCO_3_ polymorph compositions after carbonation was quantitatively analyzed by X-ray diffraction (XRD) analysis. The decalcification process of C-S-H after carbonation was carried out by employing the ^29^Si magic angle spinning nuclear magnetic resonance (^29^Si MAS NMR). The morphologies induced by carbonation were observed by scanning electron microscopy (SEM) accompanied with energy disperse spectroscopy (EDS) to confirm the chemical phases.

## 2. Materials and Methods

The cement was made by the China United Cement Corporation, and the metallurgy slag was provided by Antuoshan Corporation in Shenzhen, China. The oxide compositions of the cement and slag were given in Table 1 by X-ray fluorescence measurements (Bruker AXS, S4 Explorer, Germany).

**Figure 1:**
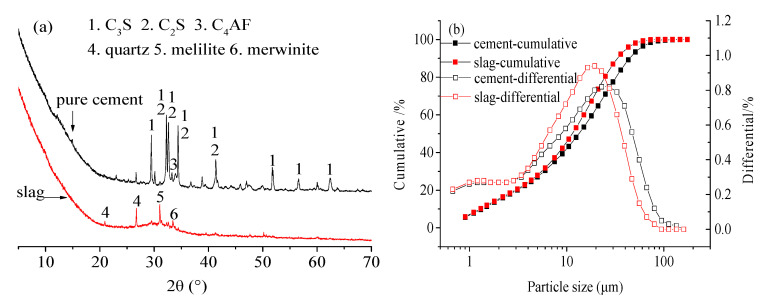
Mineral compositions and particle size distributions of cement and slag powder: (**a**) mineral compositions, (**b**) particle size distribution.

XRD results (Figure 1a) showed that cement mainly consisted of C_3_S and C_2_S, while the main minerals in slag were quartz, melilite and merwinite.

**Figure 2:**
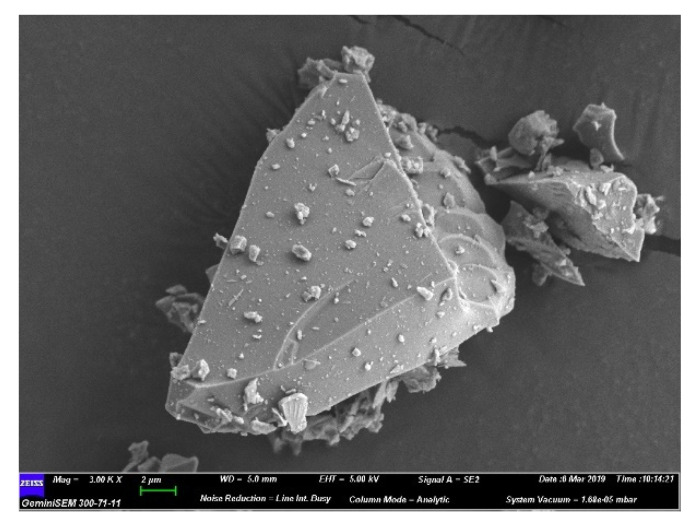
Morphology of slag particles.

The particle size distribution of raw materials is shown in Figure 1b. The typical morphology of slag (Figure 2) manifests as smooth and irregular particles.

The cement pastes were fabricated with a water-to-binder ratio of 0.56, with 20 wt.% of cement replacement by slag. The fresh pastes were cast into plastic tubes (Φ 10 mm × 100 mm) and sealed to prevent atmospheric carbonation after removing entrapped air bubbles by vibration. To prevent samples from bleeding, the samples were rotated for 24 h at a speed of 10 rpm. Afterwards, all samples were cured indoors for 60 days with the temperature at 20 °C and relative humidity (RH) at 99%. Once the samples finish curing, they were put into the carbonation chamber.

The accelerated carbonation experiment was conducted with three CO_2_ concentrations (i.e., 3, 20 and 100%). The 3% and 20% CO_2_ tests followed the European and Chinese standards for accelerated carbonation; the 100% CO_2_ test was used to study the influence of the highest possible CO_2_ concentration. During the carbonation process, the temperatures were maintained at 20 ± 2 °C, while the RH was controlled at 72 ± 2% using a saturated NaNO_3_ solution. A detailed description of the carbonation chamber was introduced in a previous paper [20]. In comparison, natural carbonation was performed under atmospheric conditions (0.03% CO_2_) with an average temperature of 25 °C and an RH of 60%.

The carbonation test was sustained and assumed to be finished at 90 days, as verified by phenolphthalein spray [23] and the stable mass of samples; however, the carbonation depth only reached to about 3 mm for the naturally carbonated sample. Therefore, for the naturally carbonated sample, only the sample within 1 mm depth from the exposed surface was used for micro measurement. The uncarbonated samples were sealed and not exposed to carbonation during the experiment process.

## 3. Analysis Tests

### 3.1. X-ray Diffraction (XRD) Analysis

The XRD was executed using a powder diffractometer (Bruker D8 Advance, Germany) with a Cu *K_α_* anode, a working voltage of 40 kV and a current of 40 mA. The cement pastes were ground to powder and sieved to less than 80 μm before measurement. The scanning program was set at 5–70° 2*θ* with a 0.02°/s step size. Qualitative and quantitative analyses of the mineral compositions were performed by EVA and TOPAS software, respectively.

### 3.2. Si Magic Angle Spinning Nuclear Magnetic Resonance (^29^Si MAS NMR) Analysis

The same samples used for XRD measurements were used for ^29^Si MAS NMR measurements. The powder was first placed into an 8 mm rotor made with ZrO_2_. Then the ^29^Si spectra, referred to Trimethylsilyl (TSPA), were collected by a spectrometer (JEOL ECZ600MHz, 14.1T, Japan) at a resonance frequency of 40 kHz and a rotational speed of 6 kHz. The scanning number and relaxation delay was 4000 times and 20 s, respectively.

The decalcification degree of C-S-H, defined as the proportions of decalcified C-S-H, can be calculated using Equation (1) [26]:

(1) $$L_{d}=\left(1-\frac{Q_{1}^{a}+Q_{2}^{a}\left(Al\right)+Q_{2}^{a}}{Q_{1}^{b}+Q_{2}^{b}\left(Al\right)+Q_{2}^{b}}\right)\times 100\%$$

where superscripts *a* and *b* represent corresponding phases after and before carbonation, respectively.

### 3.3. Scanning Electron Microscope (SEM) Observations

The microstructure of the slag blended cement pastes before and after carbonation were observed in a SEM (ZEISS Gemini, Germany) equipped with an EDS. Bulk samples were crushed and placed into a 50 °C vacuum oven for 24 h of drying. The samples’ surfaces were coated with gold before placing them into a vacuum chamber for SEM observation.

## 4. Results and Discussion

### 4.1. XRD Results

**Figure 3:**
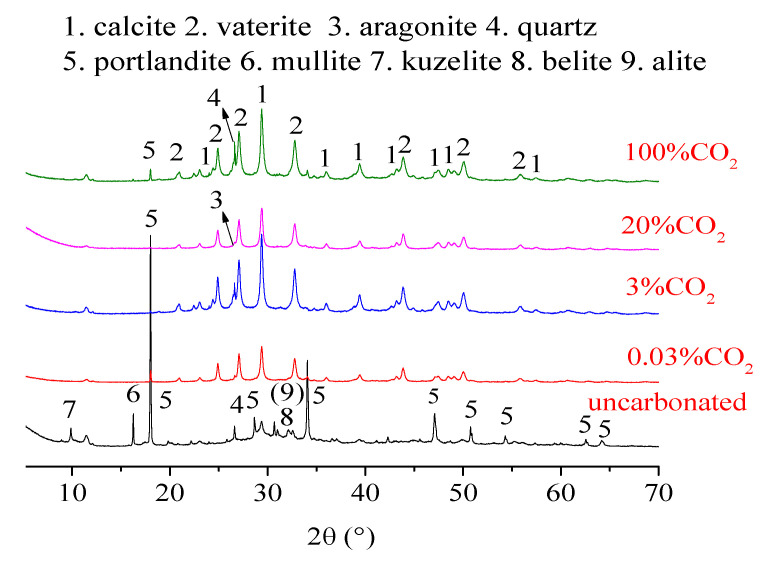
X-ray diffraction patterns of slag blended pastes with and without carbonation under different CO_2_ concentrations.

Figure 3 shows the XRD spectra acquired for the slag blended pastes with and without carbonation under different CO_2_ concentrations.

The results show that, for the uncarbonated sample, the intensity of CH was highest, indicating a relatively low degree of pozzolanic reaction after 150 days of curing. Other phases, such as larnite and kuzelite, were also detected in the uncarbonated sample.

After carbonation, the diffraction intensity of hydration productions sharply decreased with the formation of CaCO_3_. Three kinds of CaCO_3_ polymorphs (i.e., calcite, vaterite and aragonite) exist in natural environments [4,11]. In this study, calcite and vaterite were found to be the main polymorphs after carbonation of slag blended pastes, while the aragonite almost disappeared, which is consistent with previous studies [27,28,29]. Moreover, a small peak assigned to CH remained after carbonation, which may be attributed to a rather tight layer of CaCO_3_ covering the unreacted CH [30].

To study the preferential precipitation of the three polymorphs of CaCO_3_, the total content of CaCO_3_ was normalized to 100 and the relative proportions of each crystalline CaCO_3_ were quantified by TOPAS software (Table 2).

Using the ratio of c/(a + v), the effect of CO_2_ concentration on the preferential precipitation of CaCO_3_ was determined. Although the relative proportions of individual polymorphs of CaCO_3_ under different CO_2_ concentrations were not the same (Table 2), the values of c/(a + v) were almost the same under all carbonation conditions. It seems that the influence of CO_2_ concentration on the preferential precipitation of CaCO_3_ was negligible for the slag blended pastes. To explain these results, although CH is carbonated prior to C-S-H from a thermodynamic point of view [30], for the cement pastes blended with slag, abundant C-S-H generated from the pozzolanic reaction may hinder the CH in contact with the CO_2_ and to be carbonated as calcite. Therefore, the carbonation reaction in slag blended cement pastes is controlled by C-S-H instead of CH, while the allotropic precipitation of CaCO_3_ precipitated from C-S-H in slag blended pastes is not affected by CO_2_ concentration. This could explain the similar ratios of c/(a + v) under conditions of 0.03% to 100% CO_2_ and the low sensitivity of slag blended pastes to CO_2_ concentration compared to OPC and FA blended pastes.

However, by comparing the carbonation results of OPC and FA blended pastes from our previous research [20,31], in the right columns of Table 2, which has not been done by other researchers before—where cement came from the same batch—it was found that values of c/(a + v) were changed significantly with different CO_2_ concentrations. Especially, when CO_2_ concentration was elevated from 20% to 100%, c/(a + v) was further improved. Such an insistent result between slag blended pastes and OPC and FA blended pastes also suggests the low sensitivity of slag blended pastes to the CO_2_ concentration. Since the densities of the three CaCO_3_ polymorphs were different, the values of c/(a + v) should be related to the porosity after carbonation. Thus, for the slag blended pastes, the similar value of c/(a + v) indicates the similar microstructures under carbonation with different CO_2_ concentrations, while the CO_2_ concentration may change the porosities of OPC and FA blended pastes.

### 4.2. NMR Spectra

**Figure 4:**
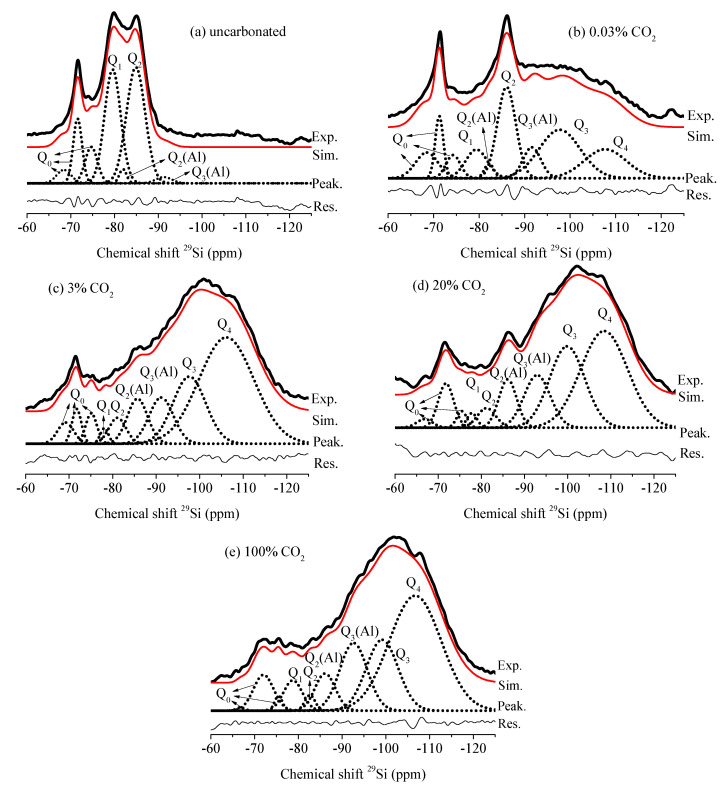
NMR spectra of slag blended cement pastes with and without carbonation: (**a**) uncarbonated; (**b**) 0.03% CO_2_; (**c**) 3% CO_2_; (**d**) 20% CO_2_; (**e**) 100% CO_2_.

The changing co-ordinations of Si atoms in C-S-H gel with carbonation effect were determined from NMR measurements, as shown in Figure 4.

The corresponding chemical shifts and relative intensities were deconvoluted by the Gaussian function and are presented in Table 3.

The chemical environments of Si atoms were expressed as *Q_n_*, where *n* represents the number of oxygen atoms connected to the SiO_4_ tetrahedron (*n* = 0, 1, 2, 3, 4). As seen in samples before carbonation, C_3_S, *β*-C_2_S and γ-C_2_S were identified by the Q_0_ peaks located from −66.8 to 75.5 ppm, while Q_1_, Q_2_ and Q_2_(Al) refer to the existence of C-S-H gel with chemical shifts from −77.8 to −86.1 ppm [32,33].

In samples after carbonation, both the dehydrated clinkers and C-S-H were consumed, resulting in a significant decrease in corresponding intensities. The peaks of Q_3_(Al) and Q_3_ appeared when two silicate chains of SiO_4_ tetrahedra became linked in space. Additionally, when calcium was totally decalcified from the C-S-H chain, the silica gel presented as Q_4_—Q_3_(Al), Q_3_ and Q_4_ were also identified as Ca-modified silica gels [34,35]. As seen from the results, decalcified C-S-H coexisted with the other phases in the carbonated sample, including unreacted clinkers, Ca-modified silica and CaCO_3_, which has been reported in the literature [20,36].

**Figure 5:**
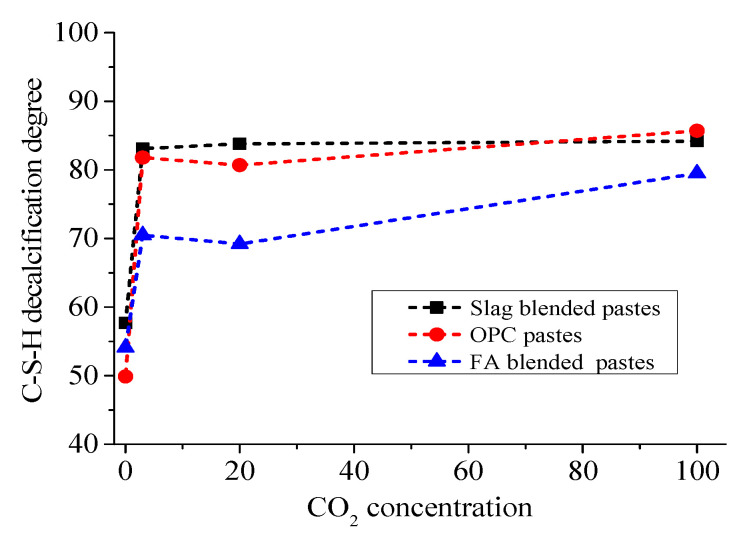
Evolution of C-S-H decalcification with the improvement in CO_2_ concentration.

The calculated decalcification degree exposed to different CO_2_ concentrations is summarized in Table 4 and depicted in Figure 5.

Figure 5 shows that the decalcification degree of C-S-H gel under 0.03% CO_2_ (57.7%) was lowest and has a similar value (around 84%) under accelerated carbonation with CO_2_ concentrations varying from 3% to 100%. In the natural atmosphere, the CO_2_ concentration is very low (about 0.03% in volume); thus, according to Fick’s second law [6], the diffusion of CO_2_ into the cement matrix is much slower than that in accelerated carbonation due to the small concentration of atmospheric CO_2_ at its interface with the surface of the concrete. When the CO_2_ reaches the interior of the concrete, it reacts with the C-S-H, and the shortage of CO_2_ in the natural condition may not be able to compensate the CO_2_ consumption in a timely manner, leading to a low decalcification degree of C-S-H after carbonation under 0.03% CO_2_. However, under accelerated conditions, the abundant supply of CO_2_ gas makes C-S-H more susceptible to carbonation, resulting in a high decalcification degree of C-S-H.

For the slag blended pastes, it was found that the degree of decalcification slowly improved from 83.1 to 84.2%, with an increase in CO_2_ concentration from 3% to 100% (Table 4 and Figure 5).

However, this improvement is too small to affect the ratios of c/(a + v), as evidenced in the XRD results. In contrast with the slag blended pastes, the decalcification degree in OPC and FA blended pastes was further improved when CO_2_ concentrations varied from 3% to 100%, as shown in Figure 5 [20,31]. That is to say, when slag is blended, the cement matrix is not as sensitive to CO_2_ concentrations as the OPC and FA blended pastes. Such a phenomenon could be attributed to the low carbonation resistance of slag blended pastes due to the lower content of CH as a buffer to resist carbonation, which, in turn, weakens the effect of the CO_2_ concentration on carbonation. For the slag blended cement pastes used in this study, high CO_2_ concentrations did not dramatically change the mineral compositions of the carbonated phases [6].

Table 4 and Figure 5 also show how, under accelerated carbonation, the C-S-H decalcification degree of slag blended and OPC pastes are similar but higher than that of FA blended pastes, indicating a lower carbonation resistance of C-S-H hydrated from slag than that hydrated from FA. Thus, when accelerated carbonation is performed, the effect of carbonation on FA blended pastes is lower than that of OPC and slag blended pastes.

### 4.3. SEM-EDS Results

**Figure 6:**
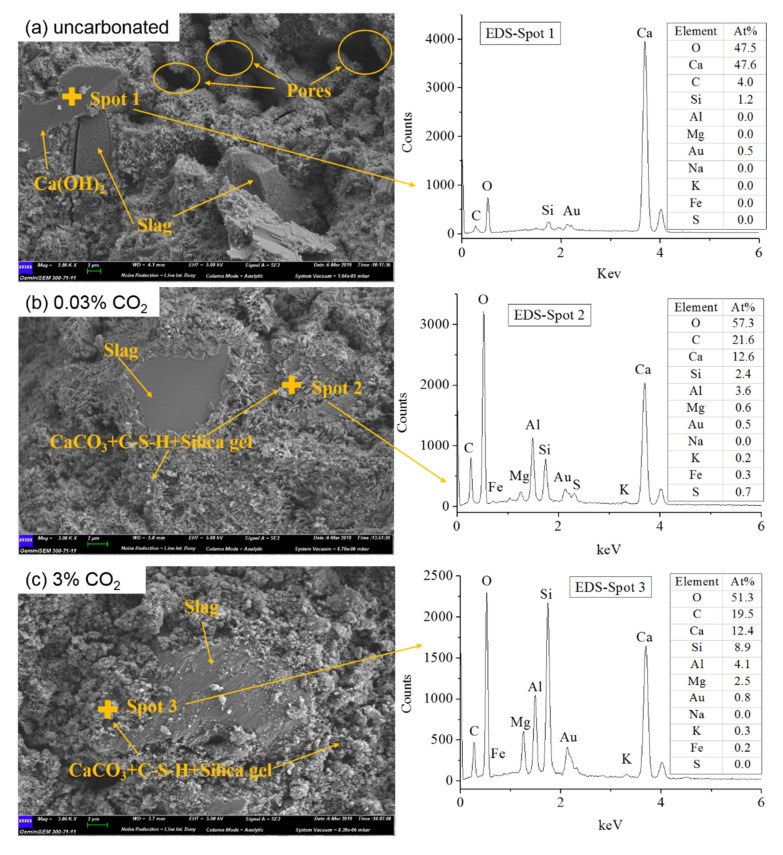

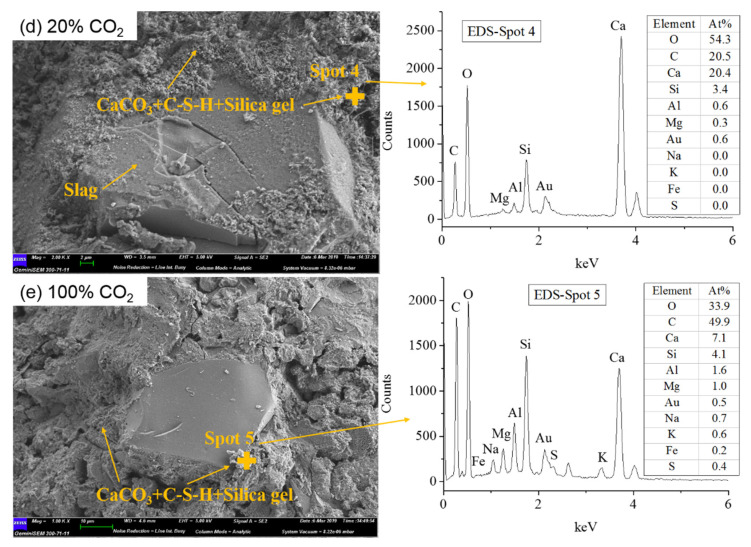
SEM images of slag blended cement pastes before and after carbonation with different concentrations: (**a**) uncarbonated, (**b**) 0.03% CO_2_, (**c**) 3% CO_2_, (**d**) 20% CO_2_, (**e**) 100% CO_2_.

Changes in the microstructures and elemental compositions of slag blended cement pastes with and without carbonation were observed by SEM-EDS, as shown in Figure 6.

Prior to carbonation, CH was observed in the SEM micrograph and evidenced by the strong peaks of Ca and O atoms in the EDS results (Figure 6a). The hydrated slag was mixed with foil-like hydration products. Before carbonation, the hardened cement paste was relatively loose, with multiple pores with diameters larger than 2 µm.

After carbonation, CH was not found in any of the SEM images (Figure 6b–e), and the foil-like hydrated phases disappeared in the images. The separate crystals of calcite, vaterite and aragonite were hard to be identified from the SEM images. Instead, the CaCO_3_ generated was found to be entangled with decalcified C-(A)-S-H and silica gel, as verified by SEM images and EDS measurements (Figure 6b–e), with simultaneous signals for Ca, O, Si and C.

Considering the effect of different CO_2_ concentrations on microstructures, the microstructure was dense but still relatively loose under natural carbonation, while it was more compact and denser under accelerated carbonation, causing a potential decrease in the transport speed of CO_2_ [12,37]. No big differences were found for the samples carbonated from 3% to 100% CO_2_, thus, it seems that the carbonated microstructure under accelerated conditions is less affected by CO_2_ concentration. Such a result is consistent with the XRD and NMR analyses that show that carbonation under CO_2_ concentrations of 3, 20 and 100% produce similar results.

Compared with the sample before carbonation, the inner unreacted and smooth slag appeared in the carbonated samples. For the sample carbonated under natural conditions, only part of the carbonated phases peeled away from the uncarbonated slag, while almost all of the unhydrated slag once again appeared on the observed section of SEM images for the samples carbonated under accelerated conditions. A probable explanation is given in following: the blended slag is difficult to hydrate completely in 60 days of curing and 90 days of carbonation, and some of the inner slag is hindered from hydration by the hydrated phases of C-S-H or C-A-S-H generated at the surface. These hydrated phases were carbonated and decalcified during the carbonation process [12,28]. As a result, the adhesion between the carbonated phases and uncarbonated slag clinker was reduced, causing the peeling of carbonated phases and the appearance of uncarbonated slag. Moreover, the low decalcification degree in natural carbonation only makes a portion of the carbonated phases peel from the carbonated slag-cement matrix, while an abundance of CO_2_ under accelerated conditions can totally destroy the adhesive ability of the carbonated phases.

## 5. Conclusions

For the slag blended cement paste, CaCO_3_ precipitated after carbonation with a dominance of vaterite and calcite polymorphs and a small amount of aragonite. The ratios of c/(a + v) under all carbonation conditions were almost the same.

The decalcification degree of C-S-H under accelerated carbonation was much higher, as compared to that under natural carbonation, while that under accelerated conditions slowly increased with higher concentrations of CO_2_. The CO_2_ concentration applied for accelerated carbonation had a slight effect on the decalcification degree of C-S-H.

According to the SEM results, more compact and denser microstructures were observed in samples under accelerated carbonation. Once the CO_2_ concentration exceeded 3%, differences in morphology were relatively modest.

In conclusion, for the cement pastes blended with 20% slag, the carbonation results under natural carbonation were different from those under accelerated carbonation. The mineral compositions and microstructures were not significantly affected by the CO_2_ concentration under accelerated carbonation, which is to say, the sensitivity of the slag blended pastes to changes in CO_2_ concentration was reduced. For the slag blended pastes, a high CO_2_ concentration can be used for accelerated carbonation without significantly changing the microstructure, compared with a low CO_2_ concentration.

## Figures and Tables

**Table 1:** Chemical composition of cement and slag.

Component	SiO_2_	Al_2_O_3_	Fe_2_O_3_	CaO	MgO	SO_3_	Na_2_O	K_2_O	Loss
Cement	20.89	5.1	2.98	64.94	1.76	3.36	0.16	0.81	1.22
Slag	31.54	13.6	0.7	41.22	9.11	2.84	0.47	0.52	-

**Table 2:** Relative proportions of CaCO_3_ polymorphs identified by TOPAS software (c = calcite; a = aragonite; v = vaterite).

Sample	OPC + Slag				OPC [20]	OPC + FA [31]
	c	a	v	c/(a + v)	c/(a + v)	c/(a + v)
0.03% CO_2_	33.4	2.2	64.4	0.5	0.77	0.51
3% CO_2_	33.1	0.2	66.7	0.5	0.30	0.64
20% CO_2_	32.2	1.8	66	0.48	0.28	0.58
100% CO_2_	32.4	3.2	64.4	0.48	0.51	0.79

**Table 3:** Deconvolution results of NMR spectra.

Deconvolved Peak	Peak Location (ppm)	Uncarbonated	0.03% CO_2_	3% CO_2_	20% CO_2_	100% CO_2_
Q_0_	−66.8 to −75.5	22.4	18.6	8.7	8.9	6.9
Q_1_	−77.8 to −79.6	33.7	8.2	1.1	1.3	4.6
Q_2_(Al)	−81.1 to −82.6	1.9	1.8	3.8	3.2	1.3
Q_2_	−84.8 to −86.1	40.2	22.1	7.9	7.8	6.1
Q_3_(Al)	−91.2 to −92.9	1.8	7.3	9.7	12.4	15.0
Q_3_	−97.6 to −99.9	0.0	26.1	18.9	25.7	18.1
Q_4_	−106.1 to −108.5	0.0	15.9	49.9	40.7	48.0

**Table 4:** Degree decalcification of C-S-H (*L_d_*) calculated from Equation (1).

CO_2_ Concentrations	*L_d_* (%)	*L_d_* (%) (OPC Pastes [20])	*L_d_* (%) (FA Pastes [31])
Uncarbonated	0	0	0
0.03%	57.7	49.9	54.1
3%	83.1	81.8	70.5
20%	83.8	80.7	69.2
100%	84.2	85.7	79.5
